# The *Tara* Oceans Project: New Opportunities and Greater Challenges Ahead

**DOI:** 10.1016/j.gpb.2015.08.003

**Published:** 2015-11-04

**Authors:** Houjin Zhang, Kang Ning

**Affiliations:** School of Life Science and Technology, Huazhong University of Science and Technology, Wuhan 430074, China

In the May 22, 2015 issue of *Science* magazine, several articles (including the editorial, research papers, and perspectives) were published on the *Tara* Oceans Project and studies related to ocean microbes [1], [2], [3], [4], [5], [6], [7], [8]. This represents truly a milestone for studies in both ocean ecology and microbial communities. As stated on the European Molecular Biology Laboratory (EMBL) website (http://www.embl.de/tara-oceans/start/): “*Tara* Oceans results reveal climate change insights, and a treasure trove of novel species and genes.”

The *Tara* Oceans project is led by the EMBL and was initiated in September 2009. Over the past few years, this project has collected more than 30,000 samples containing millions of small organisms from more than 200 ocean stations. Most samples were collected by *Tara*, a 110-foot research schooner that has sampled microscopic plankton at depths of up to 2000 m in all the major oceanic regions, such as the North Atlantic Ocean, during expeditions from 2009 through 2013 [8]. This project has garnered interest from numerous research groups worldwide, and received press coverage on several occasions since 2009 [9].

Three major studies related to microbial big-data mining have been performed on the samples and data collected in the *Tara* Oceans project. The first is a study examining the ocean microbial function and community structure [8]. The group led by Peer Bork analyzed 7.2 TB of metagenomic data (a very small fraction of the whole project) from 243 *Tara* Oceans samples collected from 68 locations. They discovered millions of novel sequences from viruses, prokaryotes, and picoeukaryotes, and indicated that the temperature, rather than other environmental factors, plays the most important role in shaping the microbial community compositions. The second study is of the ocean virome [3]. Brum et al. defined the upper-ocean viral community pan and core gene sets based on 43 *Tara* Oceans expedition samples. Their analyses support the seed-bank hypothesis, which defines “the viral community as a reservoir of dormant individuals that can potentially be resuscitated in the future under different environmental conditions” [10]. This hypothesis may help explain how oceanic viral communities maintain high local diversity. The third study is of ocean plankton interactome interpretation. Lima-Mendez et al. found that the ocean plankton interaction network is driven by both local and global patterns. Moreover, this study provided a rich resource for ocean food webs as well as biological components in ocean models [4]. Other studies published include analyses of the diversity of eukaryotic plankton [1], overview of the global ocean microbiome based on the *Tara* Oceans project [2], and the ways environmental characteristics of Agulhas rings affecting inter-ocean plankton transport [5].

## Opportunities for in-depth metagenomic analysis

Massive datasets have been generated from the *Tara* Oceans project. More than 500 high quality samples have been generated with data amounting to more than 70 TB [2], [8]. These datasets have provided great opportunities for data mining.

Firstly, the analyses of these data will help us obtain the overall picture of the microbial community structure profiles in the ocean ecosystem. Comparison of these data with those from other ocean microbial projects [11] would help to understand the dynamic nature of marine microbial communities that echo their environments.

Secondly, the whole-genome sequencing (WGS) data would provide a rich source for boosting various metagenomic assembly, functional prediction, and interaction interpretation studies [12]. Moreover, based on these routine analyses, known pathways would serve as subjects for further studies, including those related to phosphorus cycling, sulfur utilization, and nitrogen fixation.

Thirdly, to cope with such a large amount of data for analysis and to obtain meaningful interpretations, various statistical methods have been used. For instance, Spearman’s correlation coefficients and Mantel tests were used to examine the correlation between the community composition and environmental factors (meta-data for microbial communities) [2]. The results have indicated that in the surface layer, the temperature and dissolved oxygen are the predominant drivers, whereas salinity contributes to a lesser extent to shaping the structure of microbial communities [2]. Additionally, principal coordinate analysis (PCoA) of the community composition dissimilarities (Bray-Curtis) has shown that sampling locations have exerted a significant impact on community composition [2]. Furthermore, for viral community analysis, Shannon’s H index was used to quantify the diversity of the viral community, suggesting that viral genes were dispersed throughout the viral communities sampled in the *Tara* Oceans project [3].

Fourthly, analyses of these WGS data have already shed light on metabolic pathways involved in biogeochemical cycles at the sampling sites. Therefore, comparing samples from different locations will help us determine how those pathways interact with their surrounding environments. In particular, certain pathways should be present for the utilization of compounds enriched in the surrounding environment. For example, key genes encoding components in photosynthesis pathways may be plentiful in the samples collected at the surface layers, while these genes would be less abundant in samples at the deeper layer of the ocean. Another focus would be the biosynthetic gene cluster (BGC) discovery and metabolite discovery, similar to those studies of human microbial communities [13].

Finally, WGS data could be a good source for investigating individual proteins that may have unique functions [14] or sequence compositions, which are not present in their counterparts on land [15]. Subsequent experiments will therefore help to reveal mechanisms underlying the function of these proteins. Moreover, data mining may be performed to identify various biocatalysts, such as esterases, lipases, glycoside hydrolases, lactonases, and oxido-reductases, which may have broad applications in industry, such as fermentation and environmental remediation [16].

## Challenges for ocean microbial analysis

Despite the potential opportunities for in-depth metagenomic analysis, there are several hurdles that lie ahead. Firstly, accessing such massive data efficiently remains a challenge. Currently, the *Tara* Oceans data are available at three websites: the Pangea sample registry at the University of Bremen (http://doi.pangaea.de/10.1594/PANGAEA.840721), the European Nucleotide Archive (ENA, http://www.ebi.ac.uk/ena/data/view/PRJEB402), and the Metagenomics Portal at EMBL-EBI (https://www.ebi.ac.uk/metagenomics/projects/ERP001736).

The Pangea sample registry contains information such as sample codes, coordinates of the sampling sites, the date, and time of sampling. It serves as a convenient guide to sift through large amounts of data collated from the *Tara* Oceans project. However, only one link for each sample is present in the registry, which connects to the data (including sequencing data) on the ENA website.

ENA provides more data than the Pangea sample registry. As stated in the description of the ENA website, the registry was set up to list the selected set of samples published in a special issue of the *Science* magazine [8]. However, other data mentioned in these papers, such as those pertaining to the meta-transcriptome and single-cell sequencing, are not included in the Pangea sample registry.

Results from a part of the *Tara* Oceans data are stored at the Metagenomics Portal at EMBL-EBI, which include gene predictions, assemblies, in addition to the annotated Ocean Microbial Reference Gene Catalog. Both raw data and processed data, such as the predicted coding sequence (CDS) and the predicted open reading frame (ORF), are available at the website. Nevertheless, the organization of these files is not ideal: files that are listed as the predicted CDS, predicted ORF without annotation, and predicted CDS without annotation do not contain the predicted CDS or ORF. Instead, they contain the short reads that are presumably used to predict the CDS or ORF. To extract information from these files, the users have to perform a further prediction step with these files, which is also inconvenient.

Secondly, processing such large datasets more efficiently remains difficult. All raw sequencing data from different sampling sites of the *Tara* Oceans project are provided as separate files. A text file containing the links for sequences is provided at the website for each dataset. This is helpful when the users are only interested in certain sampling sites. However, if research is being conducted at a whole-project scale, it becomes cumbersome to download the files from hundreds of entries for the raw data. Even if these data are downloaded successfully, storage of such large datasets still poses a challenge for researchers without top-of-the-line storage facilities, considering its needs for storage and processing power for computation.

Thirdly, how to gain a deep understanding of the ocean ecosystem from these samples is still a bottleneck. Because metagenomic data as well as environmental meta-data are highly heterogeneous, and are usually generated from different sources, optimized statistical model are needed for reliable inference from these data. However, such models are largely lacking.

Finally, how can the *Tara* Oceans project guide us for further investigation of the oceans’ microbial communities? Based on data and initial analysis results, various experiments can be designed to investigate the marine ecosystem. Moreover, the selected sampling sites may need to be revisited at different time points of the year, and data from those visits could be analyzed to examine temporal variation of microbial communities. Only by these means, we could evaluate the effects of environmental factors, such as climate change and pollution on the oceans’ microbial communities. However, the problem lays in the cost and experimental design for such studies, which might remain difficult in the long run.

## Other indications from the *Tara* Oceans project

Studies resulting from the *Tara* Oceans project have also hinted at the profound effects of long-term investigations on the microbial diversity. From 2003 to 2007, phases I and II of the Global Ocean Sampling expedition (GOS) project, both conducted by Craig Venter, have been great hits in the ocean microbial ecosystem research area [17], [18], [19], [20], [21]. Since then, numerous studies have been conducted based on the GOS datasets, as well as new projects targeting ocean microbial diversities. These studies have provided more insights into the structural and functional properties of global ocean microbial communities [6], [7], [9]. Among them, the *Tara* Oceans project is another peak. It is only after more than a decade’s study that we are at a point, where ocean microbial communities are being decoded at a global scale, highlighting the importance of long-term investigations. Additionally, we foresee another leap forward in the area of ocean microbial ecosystem thanks to the deeper analysis of the *Tara* Oceans project data, as well as more data generated from the *Tara* Oceans project.

To summarize, the first batch of papers and datasets released by the *Tara* Oceans project have already been a great hit in the research community. Therefore, making the most of such datasets would push toward a better understanding of the ocean ecosystem. Such ocean microbial big-data would also help build novel models and methods for microbial community analyses. Nonetheless, we should also take note of the limitations present in data sharing efficiency, lack of hardware and software for biological big-data analyses, and more importantly, our inability to develop advanced data-to-knowledge strategies for largely-unknown subjects, such as ocean microbial communities. As such, important information of these ocean microbial communities remains hidden in these data, waiting to be uncovered through continuous investigation.

## Competing interests

The authors declare that they have no competing interests.
